# Mpox virus OPG175 negatively regulates viral replication by controlling Wnt signaling

**DOI:** 10.1016/j.isci.2025.114105

**Published:** 2025-11-19

**Authors:** Yoshitaka Nakata, Masako Yamasaki, Yukio Watanabe, Keiya Uriu, Rina Hashimoto, Takuya Yamamoto, The Genotype to Phenotype Japan (G2P-Japan) Consortium, Kei Sato, Akatsuki Saito, Kazuo Takayama

**Affiliations:** 1 Department of Synthetic Human Body System, Medical Research Laboratory, Institute of Integrated Research, Institute of Science Tokyo, Tokyo 113-8510, Japan; 2 Center for iPS Cell Research and Application (CiRA), Kyoto University, Kyoto 606-8507, Japan; 3 Division of Systems Virology, Department of Microbiology and Immunology, The Institute of Medical Science, The University of Tokyo, Tokyo 108-8639, Japan; 4 Medical-risk Avoidance based on iPS Cells Team, RIKEN Center for Advanced Intelligence Project (AIP), Kyoto 606-8507, Japan; 5 Institute for the Advanced Study of Human Biology (WPI-ASHBi), Kyoto University, Kyoto 606-8303, Japan; 6 Graduate School of Medicine, The University of Tokyo, Tokyo 113-8654, Japan; 7 Graduate School of Frontier Sciences, The University of Tokyo, Kashiwa 277-0882, Japan; 8 International Research Center for Infectious Diseases, The Institute of Medical Science, The University of Tokyo, Tokyo 108-8639, Japan; 9 International Vaccine Design Center, The Institute of Medical Science, The University of Tokyo, Tokyo 108-8639, Japan; 10 Collaboration Unit for Infection, Joint Research Center for Human Retrovirus infection, Kumamoto University, Kumamoto 860-0811, Japan; 11 Faculty of Medicine, Chulalongkorn University, Bangkok 10330, Thailand; 12 Department of Veterinary Science, Faculty of Agriculture, University of Miyazaki, Miyazaki 889-2192, Japan; 13 Graduate School of Medicine and Veterinary Medicine, University of Miyazaki, Miyazaki 889-1692, Japan; 14 Center for Animal Disease Control, University of Miyazaki, Miyazaki 889-2192, Japan

**Keywords:** immunology, virology, transcriptomics

## Abstract

Mpox virus (MPXV) infection exhibits different case fatality rates and symptoms depending on its clade. However, there has been insufficient molecular biological analysis of the differences between clades. Here, we investigated whether clades can be distinguished by focusing on the expression of *MPXV* genes. The replication efficiency of MPXV clade IIb, responsible for the 2022 mpox outbreak, was lower than that of clades Ia and IIa. We found that OPG175 was highly expressed in MPXV clade IIb-infected cells. Suppression of OPG175 expression significantly increased the infectious titer of MPXV, whereas OPG175 overexpression significantly decreased it. We also found that OPG175 overexpression enhanced the expression of Wnt signaling-related genes, and activation of Wnt signaling decreased MPXV replication efficiency. Therefore, high OPG175 expression in MPXV clade IIb-infected cells likely inhibits MPXV replication via activation of Wnt signaling.

## Highlights

• OPG175 is highly expressed in MPXV clade IIb-infected cells
• OPG175 overexpression decreased MPXV infection efficiency
• OPG175 knockdown increased MPXV infection efficiency
• OPG175 overexpression activated Wnt signaling

## Graphical abstract

**Figure:**
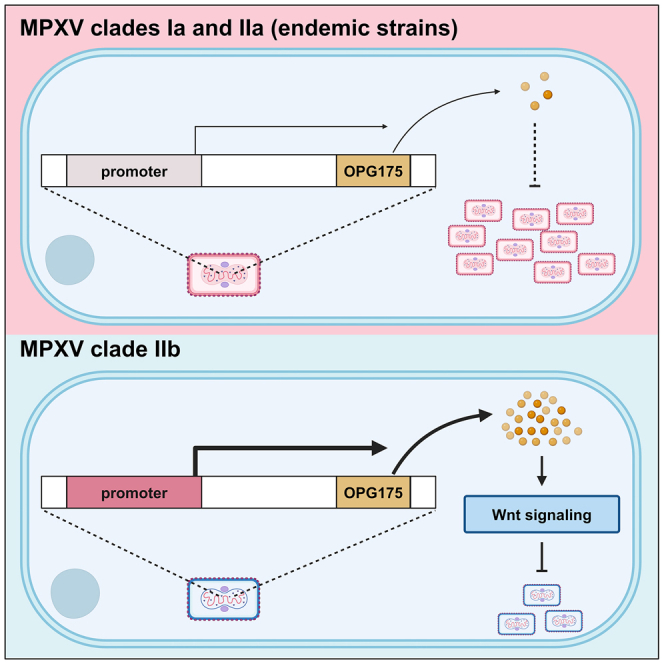

## Introduction

Mpox is an infectious disease caused by the mpox virus (MPXV), a double-stranded DNA virus. MPXV strains are classified into two major phylogenetic clades, I and II, each further divided into two subclades: Ia, Ib, IIa, and IIb. Since the first documented human infections with MPXV clade Ia in 1970,^1^ both clades Ia and IIa have become endemic in Central and West Africa.^2^^,^^3^ In 2022, an mpox outbreak caused by MPXV clade IIb occurred, leading the World Health Organization (WHO) to declare a Public Health Emergency of International Concern (PHEIC) in July of that year.^4^ Since 2023, the number of MPXV clade Ib infections has risen in the Democratic Republic of the Congo,^5^ prompting the WHO to declare another PHEIC in August 2024.^6^ The mortality rate of MPXV clade I is higher than that of clade II.^3^ Additionally, MPXV clade IIa was primarily found in Central and West Africa, whereas MPXV clade IIb spread to nonAfrican regions.^7^ Studies comparing the different MPXV clades are needed to better understand the characteristics of the MPXV responsible for outbreaks.

The MPXV genome is approximately 200 kbp in size and encodes around 190 proteins.^8^ The genome is divided into three regions: the core region, the right arm, and the left arm. The arm regions contain inverted terminal repeats (ITRs). It has been suggested that while proteins encoded in the core region are involved in viral replication and assembly, those in the arm regions are associated with pathogenicity and host range.^8^^,^^9^^,^^10^ Additionally, some MPXV proteins have been reported to be encoded exclusively by particular MPXV clades.^11^^,^^12^ Furthermore, genomic analysis of MPXV has identified several single-nucleotide polymorphisms (SNPs) specific to each clade.^13^ However, there has been insufficient analysis of MPXV genes to explain the differences in characteristics among the various MPXV clades.

We previously conducted infection experiments with MPXV clades Ia, IIa, and IIb to investigate differences among these clades.^14^ To study viral replication and host responses in human skin and colon, which are presumed primary sites of MPXV infection, we performed infection experiments using human keratinocytes and colon organoids derived from human-induced pluripotent stem (iPS) cells. We found that all MPXV clades replicated more efficiently in keratinocytes than in colon organoids. Interestingly, the replication efficiency of MPXV clade IIb was lower than that of clades Ia and IIa. However, the MPXV genes responsible for these differences among clades remain unknown.

In this study, we analyzed the expression profiles of MPXV genes in cells infected with different clades. We focused on MPXV genes with higher expression in MPXV clade IIb-infected cells than in cells infected by endemic MPXV strains, aiming to clarify the virological properties of MPXV clade IIb through functional analysis of genes highly expressed in cells infected with this clade.

## Results

### OPG175 is highly expressed in MPXV clade IIb-infected keratinocytes

**Figure 1:**
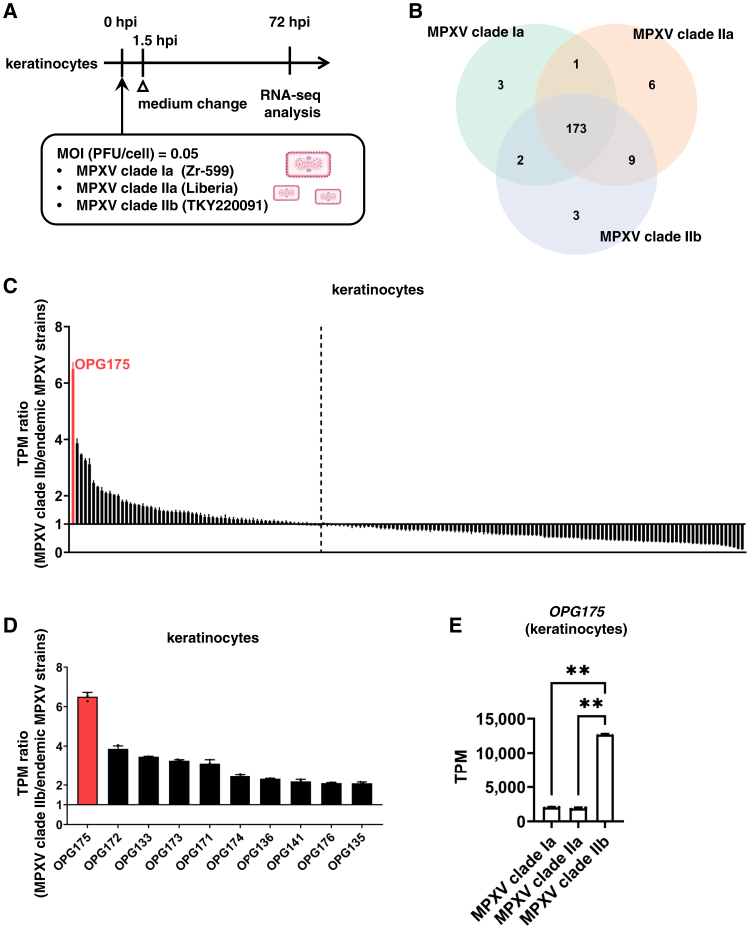
OPG175 is highly expressed in MPXV clade IIb-infected keratinocytes (A) Experimental procedure for mpox virus (MPXV) infection experiments. Keratinocytes were infected with MPXV (0.05 plaque-forming units (PFU)/cell) and cultured for 72 h. Three MPXV strains—clade Ia (Zr-599), clade IIa (Liberia), and clade IIb (TKY220091)—were used in this experiment. (B) Venn diagram showing the number of homologous proteins among MPXV clade Ia, clade IIa, and clade IIb. See also Figure S1. (C and D) Ratios of all (C) or the top 10 (D) MPXV genes in MPXV-infected keratinocytes. The expression of viral genes in MPXV clade IIb-infected keratinocytes was divided by that in endemic MPXV strain-infected keratinocytes. MPXV OPG175 is highlighted in red. Data are shown as mean + SD (*n* = 3). (E) Transcripts per kilobase million (TPM) values of *OPG175* in keratinocytes infected with MPXV clade Ia, clade IIa, or clade IIb. See also Figure S2. One-way analysis of variance (ANOVA) followed by Tukey’s post hoc test (∗∗*p* < 0.01). Data are shown as mean + SD (*n* = 3).

To characterize MPXV, we infected human keratinocytes with three MPXV strains—MPXV clade Ia (Zr-599), MPXV clade IIa (Liberia), and MPXV clade IIb (strain TKY220091)—and analyzed the infected cells by RNA-seq (Figure 1A). Cluster analysis showed that the MPXV gene expression profile in MPXV clade IIb-infected keratinocytes was distinct from that in clade Ia- or IIa-infected keratinocytes (Figure S1A). Pairwise alignment demonstrated that 173 MPXV genes were conserved among the three MPXV clades (Figures 1B, S1B, and S1C). We compared the expression of these 173 MPXV genes between MPXV clade IIb- and endemic MPXV strain-infected keratinocytes. Interestingly, the expression of orthologous poxvirus genes (OPG)171-OPG176 in MPXV clade IIb-infected keratinocytes was higher than in endemic MPXV strain-infected keratinocytes (Figures 1C and 1D). In particular, the *OPG175* gene was expressed approximately 6-fold higher in MPXV clade IIb-infected keratinocytes than in endemic strain-infected keratinocytes (Figure 1E). Similar results were observed in human iPS cell-derived colon organoids (Figure S2).

### The *OPG175* gene has superoxide dismutase-like activity

**Figure 2:**
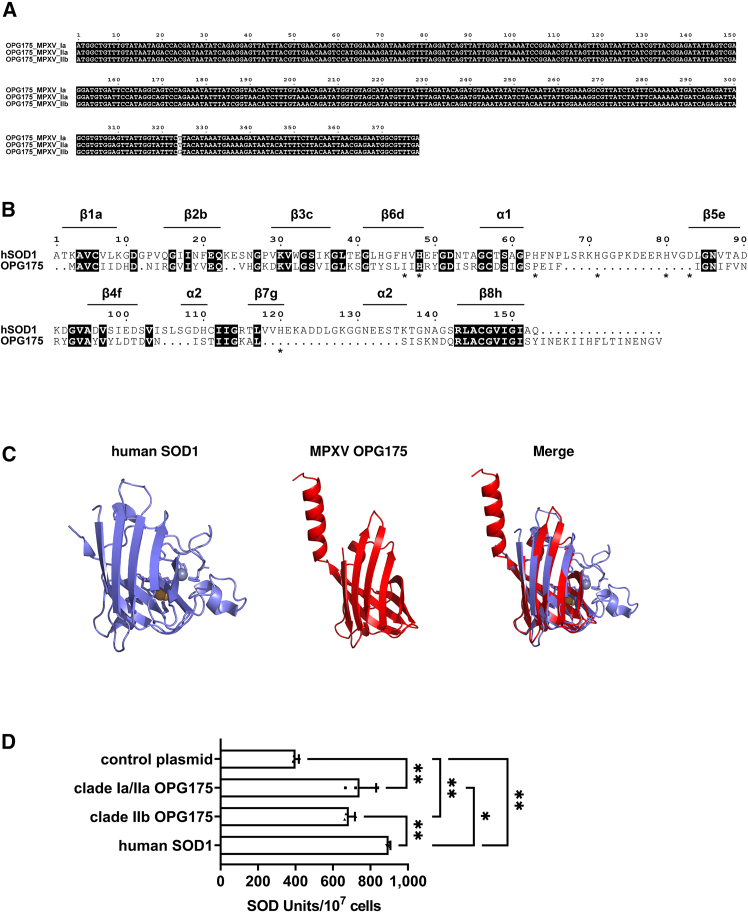
MPXV OPG175 displays SOD1-like activity (A) Multiple sequence alignment of OPG175 was performed using MEGA 11.0.13^16^ and visualized using ESPript 3.0.^17^ See also Figure S3A. (B) Amino acid sequence alignment of human superoxide dismutase (hSOD)1 and OPG175. The secondary structure of hSOD1 shown above the sequence is named according to a previous study.^18^ The amino acid residues that are identical are highlighted. Stars indicate histidine or aspartic acid residues involved in copper or zinc binding. Pairwise alignment was performed using MEGA 11.0.13 with the MUSCLE algorithm^19^ and visualized using ESPript 3.0. (C) Comparison of the hSOD1 structure (PDB: 2v0a; blue and yellow spheres indicate zinc and copper ions, respectively) with the putative structure of OPG175. The putative structure of OPG175 was generated by ColabFold,^20^ a software for predicting protein structures based on the AlphaFold2 algorithm.^21^ Each protein structure and the merged image were visualized using PyMOL 3.0.0. (D) HEK293 cells were transfected with OPG175- or hSOD1-expressing plasmids. 48 h after transfection, an SOD activity assay was performed. One-way ANOVA followed by Tukey’s post hoc test (∗*p* < 0.05, ∗∗*p* < 0.01). See also Figure S3B. Data are shown as mean + SD (*n* = 3).

We focused on the *OPG175* gene, which was expressed at a significantly higher level in MPXV clade IIb-infected cells than in endemic MPXV strain-infected cells. Multiple sequence alignment showed that the DNA sequences of the *OPG175* gene were identical among the three MPXV clades used in this study, except for one nucleotide substitution that did not result in an amino acid change (Figure 2A). We examined amino acid mutations in OPG175 using Nextstrain (https://nextstrain.org/mpox/all-clades?c=gt-nuc_148344),^15^ which analyzes diverse MPXV strains, and found that the frequency of amino acid mutations in the *OPG175* gene was low (Figure S3A). Therefore, the function of the *OPG175* gene is likely conserved among MPXV clades.

Previous studies using the vaccinia virus (VACV) have shown that *Orthopoxvirus* OPG175 is a homolog of human superoxide dismutase (hSOD) 1.^22^^,^^23^ Pairwise alignment of the amino acid sequences of hSOD1 and MPXV OPG175 showed that the sequence identity was 30.67% (Figure 2B). However, it was also found that OPG175 does not contain the amino acid sequences required for binding of copper or zinc ions to hSOD1.^24^ The structure of MPXV OPG175 predicted using AlphaFold2,^21^ especially its partial β-barrel structure, was found to be similar to that of hSOD1 (PDB: 2v0a) (Figure 2C). Superimposing the structures of MPXV OPG175 and hSOD1 using the align function of PyMOL revealed high structural similarity, with a root-mean-square deviation (RMSD) of 0.833 Å. To examine whether MPXV OPG175 has SOD activity, we measured SOD activity in HEK293 cells transfected with plasmids expressing either OPG175 or hSOD1. As a result, we found that MPXV OPG175 transfection increased intracellular SOD activity (Figures 2D and S3B), suggesting that MPXV OPG175 has hSOD-like activity. Almazán et al. reported that the recombinant protein of the OPG175 homolog encoded in VACV had no enzymatic activity,^22^ while MPXV OPG175 showed SOD-like activity in this study. Although the reason for the functional differences between OPG175 in MPXV and VACV remains unclear, it is possible that not all findings observed in VACV are applicable to MPXV.

### OPG175 inhibited MPXV replication by regulating Wnt signaling

**Figure 3:**
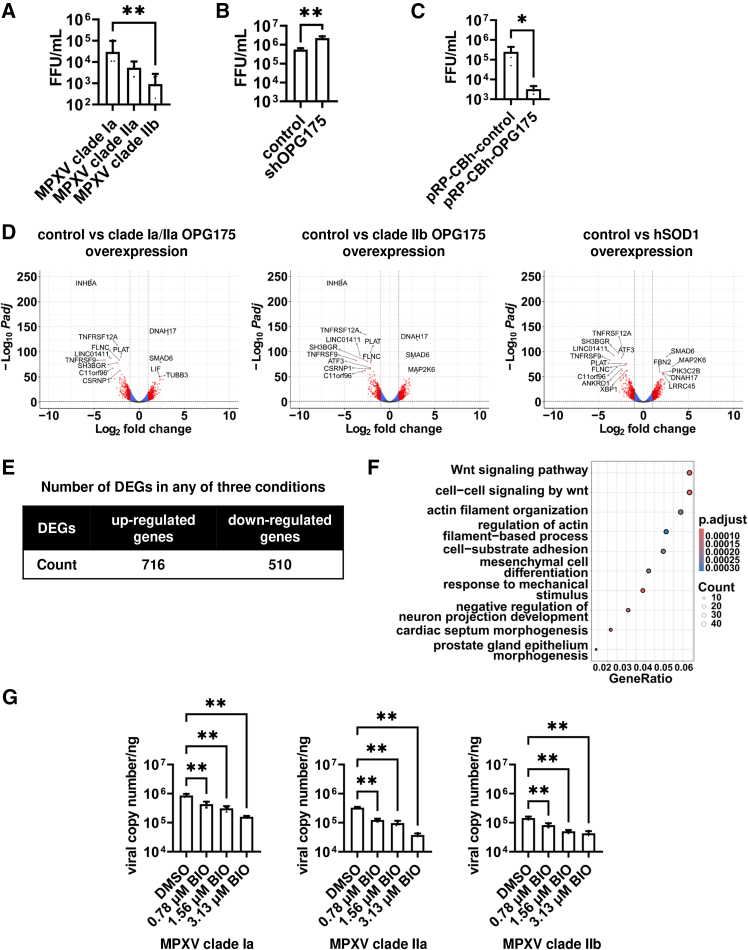
OPG175 reduces MPXV replication (A) HEK293 cells were infected with MPXV clade Ia, IIa, or IIb at 0.05 multiplicity of infection (MOI) and cultured for 96 h. Focus-forming unit (FFU) values were measured at 96 h post-infection (hpi). One-way ANOVA followed by Dunnett post hoc test (∗∗*p* < 0.01, MPXV clade IIb versus other groups). See also Figures S4A, S4B, and S5A–S5C. Data are shown as mean + SD (*n* = 4). (B) Wild-type (control) or shOPG175-expressing HEK293 cells (shOPG175) were infected with MPXV clade Ia at 0.05 MOI and cultured for 96 h. FFU values were measured at 96 hpi. Unpaired two-tailed Student’s *t* tests (∗∗*p* < 0.01). See also Figures S4C, S5D, and S5E. Data are shown as mean + SD (*n* = 4). (C) HEK293 cells were transfected with control plasmids (pRP-CBh-control) or OPG175-expressing plasmids (pRP-CBh-OPG175). One day after transfection, the cells were infected with MPXV clade Ia at 0.05 MOI and cultured for 48 h. FFU values were measured at 96 hpi. Unpaired two-tailed Student’s *t* tests (∗*p* < 0.05). See also Figures S4D and S4E. Data are shown as mean + SD (*n* = 4). (D) HEK293 cells were transfected with control, OPG175-, or hSOD1-expressing plasmids and cultured for 48 h. RNA-seq analysis was performed. Volcano plot of differentially expressed genes (DEGs) in control, OPG175-, or hSOD1-overexpressing (OE) HEK293 cells (|Log_2_ fold change| > 1.0, adjusted *p* value [*P*adj] < 0.05). (E) Number of DEGs affected by OPG175 or hSOD1 overexpression (control vs. clade Ia/IIa OPG175 OE, control vs. clade IIb OPG175 OE, or control vs. hSOD1 OE). See also Figure S6. (F) Dot plot of the top 10 significantly enriched Gene Ontology (GO) terms (Biological Process) from GO enrichment analysis of 716 upregulated genes. See also Figures S7 and S8. (G) Intracellular viral DNA in MPXV-infected HEK293 cells treated with 6-bromoindirubin-3′-oxime (BIO) at 96 hpi was measured by qPCR. One-way ANOVA followed by Dunnett’s post hoc test (∗∗*p* < 0.01, DMSO versus other groups). See also Figure S9. Data are shown as mean + SD (*n* = 3).

We measured intracellular viral DNA in MPXV-infected HEK293 cells to determine replication efficiency. The intracellular viral DNA at 2 h post infection (hpi) was equivalent among the three MPXV clades (Figure S4A), demonstrating that all clades infected the cells equally. At 96 hpi, intracellular viral DNA in MPXV clade IIb-infected cells was significantly lower compared to that of MPXV clade Ia- or IIa-infected cells (Figure S4B). In addition, the infectious viral titer of the MPXV clade IIb-infected group was significantly lower than that of MPXV clade Ia-infected group (Figure 3A). Consistent with the findings in HEK293 cells, low replication efficiency of MPXV clade IIb was also confirmed in HeLa cells (Figures S5A–S5C). These results indicate that MPXV clade IIb has lower replication efficiency than endemic MPXV strains.

Next, to examine the effects of MPXV OPG175 on MPXV replication, we performed MPXV infection experiments using HEK293 cells expressing shRNA against MPXV OPG175. Suppression of OPG175 gene expression (Figure S4C) significantly increased the infectious viral titers (Figure 3B). Similar results were obtained in HeLa cells (Figures S5D and S5E). In addition, to further investigate the influence of MPXV OPG175 on MPXV replication, we performed MPXV infection experiments using OPG175-transfected HEK293 cells. OPG175 overexpression significantly decreased intracellular viral DNA (Figures S4D and S4E) and led to a significant reduction in infectious viral titer (Figure 3C). The OPG175 knockdown and overexpression experiments suggested that MPXV OPG175 negatively regulates MPXV replication.

To investigate the effects of MPXV OPG175 expression on the host transcriptome, we transfected HEK293 cells with a plasmid expressing MPXV OPG175 and performed RNA-seq analysis. We examined differentially expressed genes (DEGs) in cells overexpressing (OE) MPXV clade Ia/IIa OPG175, MPXV clade IIb OPG175, or hSOD1 (Figure 3D). Overexpression of MPXV clade Ia/IIa OPG175, MPXV clade IIb OPG175, or hSOD1 increased the expression of 716 genes (Figure 3E), of which 349 were common among the three conditions (Figure S6). Gene Ontology (GO) enrichment analyses of these 716 genes suggested the activation of Wnt signaling (Figure 3F), with the top two enriched GO terms associated with this pathway. Cluster analysis showed that the expression profile of genes related to the canonical and noncanonical Wnt pathways was altered by overexpression of OPG175 or hSOD1 (Figure S7A). Both canonical (Figure S7B) and noncanonical (Figure S7C) Wnt signaling-related genes were significantly upregulated by OPG175 or hSOD1 overexpression. Consistent with this, the expression levels of some Wnt-related genes significantly increased in MPXV clade IIb-infected keratinocytes (Figure S8). Because hydrogen peroxide produced by the catalytic activity of SOD1 has been reported to activate Wnt signaling,^25^ it is possible that MPXV OPG175 triggers Wnt signaling through a similar mechanism.

To examine the effects of Wnt signaling activation on MPXV infection, we performed MPXV infection experiments using the Wnt pathway activator 6-bromoindirubin-3′-oxime (BIO). We confirmed that BIO treatment did not cause cytotoxicity at concentrations of 3.13 μM or lower (Figure S9A). Intracellular viral DNA was reduced in a BIO concentration-dependent manner (Figure 3G). In addition, treatment with a Wnt3A-conditioned medium also decreased the infectious viral titer (Figure S9B). These results suggest that the activation of Wnt signaling inhibits MPXV infection.

## Discussion

Here, we demonstrated that the *OPG175* gene is highly expressed in cells infected with MPXV clade IIb. Because suppression of *OPG175* gene expression increased the infectious viral titer (Figure 3B) and OPG175 overexpression decreased it (Figure 3C), the high expression of the OPG175 gene is believed to be a contributing factor to the low replication efficiency of MPXV clade IIb. In the future, to assess the clinical relevance of this finding, it will be necessary to examine correlations between viral replication efficiency and OPG175 expression using biopsy samples from individuals infected with MPXV clade IIb and endemic MPXV strains. However, since such analyses concerning differences between MPXV clades are challenging due to the limited accessibility of human specimens, animal model studies examining the replication efficiency and OPG175 expression of each MPXV clade would be a logical starting point.

We found that Wnt signaling activated by MPXV OPG175 expression negatively impacts MPXV replication efficiency. However, in this study, we could not identify the specific mechanism by which MPXV OPG175 regulates Wnt signaling. It may be possible to clarify this mechanism by analyzing the expression of Wnt receptors and other Wnt-related proteins in MPXV OPG175-OE cells. Recently, Huang et al. reported that MPXV infection alters Wnt signaling, indicating a close relationship between MPXV and Wnt signaling pathways.^26^ Wnt signaling is a crucial factor for maintaining stem cells in various organs.^27^ Therefore, further investigation into the relationship between MPXV and Wnt signaling may help illuminate the effects of MPXV infection on organ homeostasis.

In this study, we found that the MPXV OPG171-OPG176 genes were highly expressed in MPXV clade IIb-infected cells. Therefore, proteins other than MPXV OPG175 encoded in this gene cluster may affect MPXV replication efficiency. It is known that VACV OPG173 inhibits host translation initiation and suppresses innate immune responses.^28^ In addition, VACV OPG174 functions as a hydroxysteroid dehydrogenase and inhibits inflammatory responses.^29^^,^^30^ Furthermore, VACV OPG176 contains a B-cell lymphoma (Bcl)-2 domain and inhibits Toll-like receptor (TLR)- and interleukin (IL)-1 β-mediated nuclear factor-kappa B (NF-κB) activation.^31^^,^^32^ However, since these findings all relate to *VACV* genes rather than MPXV, further examination of *MPXV* genes is necessary.

### Limitations of the study

MPXV replication was significantly affected by OPG175 knockdown, OPG175 overexpression, BIO, or Wnt3A-conditioned medium, although the effects were limited. These results suggest that factors other than the OPG175 gene and Wnt signaling also play a major role in MPXV replication. Therefore, to develop effective antiviral drugs against MPXV, it will be necessary to investigate additional contributing factors.

## Resource availability

### Lead contact

Further information and requests for resources and reagents should be directed to and will be fulfilled by the lead contact, Kazuo Takayama (takayama.kazuo@tmd.ac.jp).

### Materials availability

All unique reagents generated in this study are listed in the key resources table and are available from the lead contact with a completed materials transfer agreement.

### Data and code availability

• All viral sequences used in this study were obtained from the GenBank database (https://www.ncbi.nlm.nih.gov/nuccore/). Accession IDs are listed in the key resources table. Bulk RNA-seq data from this study have been submitted to the Gene Expression Omnibus (GEO) under accession number GSE278866.
• Code: Not applicable.
• Any additional information required to reanalyze the data reported in this work is available from the lead contact upon request.

## Consortia

We thank Keita Matsuno, Naganori Nao, Hirofumi Sawa, Keita Mizuma, Jingshu Li, Izumi Kida, Yume Mimura, Yuma Ohari, Shinya Tanaka, Masumi Tsuda, Lei Wang, Yoshikata Oda, Zannatul Ferdous, Kenji Shishido, Hiromi Mohri, Miki Iida, Takasuke Fukuhara, Tomokazu Tamura, Rigel Suzuki, Saori Suzuki, Shuhei Tsujino, Hayato Ito, Jumpei Ito, Yu Kaku, Naoko Misawa, Arnon Plianchaisuk, Ziyi Guo, Alfredo, Jr. Amolong Hinay, Kaoru Usui, Wilaiporn Saikruang, Spyridon Lytras, Shusuke Kawakubo, Luca Nishimura, Yusuke Kosugi, Shigeru Fujita, Jarel Elgin Mendoza Tolentino, Luo Chen, Lin Pan, Wenye Li, Maximilian Stanley Yo, Kio Horinaka, Mai Suganami, Mika Chiba, Ryo Yoshimura, Kyoko Yasuda, Keiko Iida, Adam Patrick Strange, Naomi Ohsumi, Shiho Tanaka, Eiko Ogawa, Kaho Okumura, Ysuki Fukuda, Rina Osujo, Kazuhisa Yoshimura, Kenji Sadamasu, Mami Nagashima, Hiroyuki Asakura, Isao Yoshida, So Nakagawa, Sayaka Deguchi, Hiroki Futatsusako, Takao Hashiguchi, Tateki Suzuki, Kanako Kimura, Jiei Sasaki, Yukari Nakajima, Hisano Yajima, Takashi Irie, Ryoko Kawabata, Kaori Tabata, Terumasa Ikeda, Hesham Nasser, Ryo Shimizu, MST Monira Begum, Michael Jonathan, Yuka Mugita, Sharee Leong, Otowa Takahashi, Takamasa Ueno, Chihiro Motozono, Mako Toyoda, Anon Kosaka, Miki Kawano, Natsumi Matsubara, Tomoko Nishiuchi, Jiri Zahradnik, Prokopios Andrikopoulos, Miguel Padilla Blanco, Aditi Konar, and Ruojin Tian as the members of The Genotype to Phenotype Japan (G2P-Japan) Consortium.

## Acknowledgments

We thank Dr. Kelvin Hui (Kyoto University) for critical reading of the manuscript; Ms. Kazumi Deguchi and Ms. Satoko Sakurai (Kyoto University) for technical assistance with the RNA-seq experiments; and Ms. Natsumi Mimura, Ms. Ayaka Sakamoto (Institute of Science Tokyo), Ms. Naoko Yasuhara, and Ms. Shiho Morimoto (Kyoto University) for technical assistance. We also thank all members of The Genotype to Phenotype Japan (G2P-Japan) Consortium. Figure 1A and the graphical abstract were created using BioRender (https://biorender.com). This research was supported by the iPS Cell Research Fund, JSPS Core-to-Core Program (A. Advanced Research Networks), and the 10.13039/100009619Japan Agency for Medical Research and Development (AMED) (JP21gm1610005, JP23fk0108583, JP23jf0126002, and JP24fk0108907).

## Author contributions

Y.N. analyzed all samples and wrote the manuscript; M.Y. analyzed and interpreted the data and wrote the manuscript; Y.W. performed RNA-seq analysis; K.U. performed infection experiments; R.H. analyzed and interpreted the data and wrote the manuscript; and T.Y. performed RNA-seq analysis. The Genotype to Phenotype Japan (G2P-Japan) Consortium contributed to project administration. K.S. conceived and designed the study. A.S. performed infection experiments and wrote the manuscript. K.T. conceived and designed the study, analyzed and interpreted the data, and wrote the manuscript.

## Declaration of interests

K.S. has received consulting fees from Moderna Japan Co., Ltd., and Takeda Pharmaceutical Co. Ltd., and honoraria for lectures from Gilead Sciences, Inc., Moderna Japan Co., Ltd., and Shionogi & Co., Ltd. The other authors have no conflicts of interest to report.

## STAR★Methods

### Key resources table

**Table:** 

REAGENT or RESOURCE	SOURCE	IDENTIFIER
**Antibodies**		

Mouse anti-Vaccinia virus L1 protein antibody	BioAcademia	Cat# 65-038 RRID: N/A
Goat anti-mouse IgG (H+L)-HRP	KPL	Cat# 5220-0341 RRID: AB_2891080

**Bacterial and virus strains**		

MPXV clade Ia (Zr-599)	provided by National Institute of Infectious Diseases, Japan	NC_003310.1
MPXV clade IIa (Liberia)	provided by National Institute of Infectious Diseases, Japan	DQ011156.1
MPXV clade IIb (TKY220091)	provided by Tokyo Metropolitan Institute of Public Health, Japan	LC722946.1

**Chemicals, peptides, and recombinant proteins**		

KGM-Gold Keratinocyte Growth Medium BulletKit	Lonza	Cat# 00192060
Human laminin 511 E8 fragments (iMatrix-511)	Nippi	Cat# 892 012
StemFit AK02N medium	Ajinomoto Healthy Supply	Cat# RCAK02N
Y-27632	FUJIFILM Wako Pure Chemical	Cat# 034-24024
TrypLE Select Enzyme	Thermo Fisher Scientific	Cat# 12563029
Matrigel® Growth Factor Reduced Basement Membrane	Corning	Cat# 354230
Activin A	R&D Systems	Cat# 338-AC-01M
RPMI1640 medium	Sigma-Aldrich	Cat# R8758-500
B-27 Supplement Minus Vitamin A	Thermo Fisher Scientific	Cat# 12587001
GlutaMAX	Thermo Fisher Scientific	Cat# 35050-079
Penicillin−streptomycin	Nacalai Tesque	Cat# 26239-42; Cat# 32204-92
FGF2	Katayama Chemical Industries	Cat# 160-0010-3
CHIR99021	FUJIFILM Wako Pure Chemical	Cat# 034-23103
A-83-01	FUJIFILM Wako Pure Chemical	Cat# 035-24113
Noggin	PeproTech	Cat# 120-10C
Forskolin	FUJIFILM Wako Pure Chemical	Cat# 063-02193
EGF	PeproTech	Cat# AF-100-15
N2	FUJIFILM Wako Pure Chemical	Cat# 141-08941
Dulbecco’s Modified Eagle Medium with L-Glutamine and Phenol Red	FUJIFILM Wako Pure Chemical	Cat# 044-29765
Fetal bovine serum	Corning	Cat# 35-010-CV
DMEM (high glucose)	Sigma-Aldrich	Cat# 6429-500ML
Fetal bovine serum	Sigma-Aldrich	Cat# 172012-500ML
Penicillin−streptomycin	Sigma-Aldrich	Cat# P4333-100ML
DMEM (low glucose)	Sigma-Aldrich	Cat# D6046-500ML
Paraformaldehyde phosphate	Nacalai Tesque	Cat# 09154-85
Methylene blue	Nacalai Tesque	Cat# 22412-14
Formaldehyde neutral buffer solution	Nacalai Tesque	Cat# 37152-51
Triton X-100	Nacalai Tesque	Cat# 35501-15
Metal Enhancer for DAB Stain	Nacalai Tesque	Cat# 07388-24
TaqMan Fast Advanced Master Mix for qPCR	Thermo Fisher Scientific	Cat# 4444557
ISOGEN	NIPPON GENE	Cat# 319-90211
SYBR Green PCR Master Mix	Thermo Fisher Scientific	Cat# 4385614
PEI Max	Polysciences	Cat# 24765-1
Lipofectamine 2000	Thermo Fischer Scientific	Cat# 11668019
Blasticidin S	Thermo Fisher Scientific	Cat# 03759-71
Hygromycin B	Nacalai Tesque	Cat# 09287-84
Puromycin	Thermo Fisher Scientific	Cat# 14861-71
6-Bromoindirubin-3'-oxime	Sigma-Aldrich	Cat# 361550
Lipofectamine RNAiMAX	Thermo Fischer Scientific	Cat# 13778150

**Critical commercial assays**		

Peroxidase Stain 3,3’-diaminobenzidine (DAB) Kit	Nacalai Tesque	Cat# 25985-50
DNeasy Blood & Tissue Kit	QIAGEN	Cat# 69506
Superscript VILO cDNA Synthesis Kit	Thermo Fisher Scientific	Cat# 11754250
SOD Assay Kit - WST	DOJINDO LABORATORIES	Cat# S311
Cell Counting Kit-8	FUJIFILM Wako Pure Chemical	Cat# 347-07621
Stranded mRNA Prep Kit	Illumina	Cat# 20040532

**Deposited data**		

Crystal structure of human superoxide dismutase	Strange et al.^18^	PDB: 2v0a
GENCODE (release 32, GRCh38.p13)	Frankish et al.^33^	https://www.gencodegenes.org/human/release_32.html
RNA-seq data generated from OPG175 and hSOD1 overexpression experiments	This paper	GSE278866
RNA-seq data of MPXV-infected keratinocytes or human iPS cell-derived colon organoids	Watanabe et al.^14^	GSE219036

**Experimental models: Cell lines**		

Normal Human Epidermal Keratinocytes-Adult	Lonza	Cat# 00192627
Human: iPS cells (1383D6)	provided by Dr. Masato Nakagawa, Kyoto University	N/A
HEK293 cells	JCRB Cell Bank	Cat# JCRB9068
HEK293 cells	ATCC	Cat# CRL-1573
African green monkey (*Chlorocebus sabaeus*): VeroE6 cells	provided by Tokyo Metropolitan Institute of Public Health, Japan	N/A
L Wnt-3A cells	ATCC	Cat# CRL-2647
HeLa cells	RIKEN BRC	Cat# RCB0007

**Oligonucleotides**		

Primers and probe for the quantification of viral DNA copy number of the cell: Forword: GGAAAATGTAAAGACAACG AATACAG Reverse: GCTATCACATAATCTGGAA GCGTA Probe: FAM-AAGCCGTAATCTATGTTG TCTATCGTGTCC-BHQ1	Li et al.^34^	N/A
Primers used for RT-qPCR, See Table S1	This paper	N/A
siRNA used for knockdown experiments, See Table S3	This paper	N/A
Negative Control DsiRNA	Integrated DNA Technologies	51-01-14-04

**Recombinant DNA**		

pRP[Exp]-CBh>[MPXV I gp152]	VectorBuilder	VB230516-1089snp
pRP[Exp]-CBh>[MPXV IIb BDQ10531.1]	VectorBuilder	VB230516-1094nak
pRP[Exp]-CBh>hSOD1	VectorBuilder	VB230516-1107bkv
pRP[Exp]-CBh>ORF_Stuffer	VectorBuilder	VB230622-1540udv
pPB[shRNA]-Bsd-U6>[clade_IIb_OPG175_105-127]	VectorBuilder	VB231109-1138pwv
pPB[shRNA]-Hygro-U6>[clade_IIb_OPG175_215-237]	VectorBuilder	VB231109-1137pdb
pPB[shRNA]-Puro-U6>[clade_IIb_OPG175_239-261]	VectorBuilder	VB231109-1136hjg
pHL-EF1a-hcPBase	provided by Dr. Akitsu Hotta, Kyoto University	N/A
pCMV-EGFP	NEPA GENE	N/A

**Software and algorithms**		

MEGA 11.0.13	Tamura et al.^16^	https://www.megasoftware.net
ESPript 3.0	Robert et al.^17^	https://espript.ibcp.fr/ESPript/ESPript
ColabFold	Mirdita et al.^20^	https://github.com/sokrypton/ColabFold
PyMOL 3.0.0	Schrödinger	https://www.pymol.org
siDirect	Naito et al.^35^^,^^36^	http://sidirect2.rnai.jp
Cutadapt ver v4.6	Martin et al.^37^	https://pypi.org/project/cutadapt
STAR ver 2.7.11a	Dobin et al.^38^	https://github.com/alexdobin/STAR
htseq-count ver. 2.0.5	Anders et al.^39^	https://pypi.org/project/HTSeq
R v4.4.0	The R foundation	https://www.r-project.org
DESeq2 v1.42.0	Love et al.^40^	https://bioconductor.org/packages/release/bioc/html/DESeq2.html
clusterProfiler v4.12.0	Wu et al.^41^ Yu et al.^42^	https://bioconductor.org/packages/release/bioc/html/clusterProfiler.html
ComplexHeatmap v2.20.0	Gu et al.^43^	https://bioconductor.org/packages/release/bioc/html/ComplexHeatmap.html
Prism 9 software v9.5.1	GraphPad Software	https://www.graphpad.com/scientific-software/prism
BioRender	BioRender	https://www.biorender.com

**Other**		

96-well cell culture plates	Thermo Fisher Scientific	Cat# 167008
Collagen I-coated 96-well cell culture plates	AGC TECHNO GLASS	Cat# 4860-010
BioCoat® Poly-D-Lysine 96-well Clear Flat Bottom TC-treated Microplate, with Lid	Corning	Cat# 356461
Nextstrain	Hadfield et al.^15^	https://nextstrain.org/mpox/all-clades
QuantStudio 3 real-time PCR system	Thermo Fisher Scientific	N/A
StepOnePlus real-time PCR system	Thermo Fisher Scientific	N/A
Multiskan FC	Thermo Fisher Scientific	N/A
4D-Nucleofector system	Lonza	N/A
2100 Bioanalyzer	Agilent Technologies	N/A
NextSeq2000	Illumina	N/A
IX-83	Evident Corporation	N/A

### Experimental model and study participant details

#### Normal human epidermal keratinocytes

Normal Human Epidermal Keratinocytes-Adult (NHEK-Ad, Cat# 00192627, Lonza) were cultured with KGM-Gold Keratinocyte Growth Medium BulletKit (Cat# 00192060, Lonza). NHEK-Ad were seeded on 24-well cell culture plates (2.0×10^4^ cells/2 cm^2^) and cultured for 7 days.

#### Human iPS cell-derived colon organoids

The human induced pluripotent stem (iPS) cell line, 1383D6, was maintained on 0.5 μg/cm^2^ recombinant human laminin 511 E8 fragments (iMatrix-511, Cat# 892 012, Nippi) with StemFit AK02N medium (Cat# RCAK02N, Ajinomoto) containing 10 μM Y-27632 (Cat# 034-24024, FUJIFILM Wako Pure Chemical). For passaging, human iPS cell colonies were treated with TrypLE Select Enzyme (Cat# 12563029, Thermo Fisher Scientific) for 10 min at 37°C. After centrifugation, cells were seeded on Matrigel® Growth Factor Reduced Basement Membrane (Cat# 354230, Corning)-coated cell culture plates (2.0 × 10^5^ cells/4 cm^2^) and cultured for 2 days. To perform definitive endoderm differentiation, human iPS cells were treated with 100 ng/mL Activin A (Cat# 338-AC-01M, R&D Systems) and 10 μM Y-27632 in RPMI1640 medium (Cat# R8758-500, Sigma-Aldrich) supplemented with 1× B-27 Supplement Minus Vitamin A (Cat# 12587001, Thermo Fisher Scientific), 1× GlutaMAX (Cat# 35050-079, Thermo Fisher Scientific), and 1× penicillin−streptomycin (Cat# 26239-42, Cat# 32204-92, Nacalai Tesque) for 3 days. To perform hindgut differentiation, cells were treated with 200 ng/μL FGF2 (Cat# 160-0010-3, Katayama Chemical Industries) in RPMI1640 medium supplemented with 1× B-27 Supplement Minus Vitamin A, 1× GlutaMAX, and 1× penicillin−streptomycin for 4 days. To generate colon organoids, cells were dissociated and embedded in Matrigel Growth Factor Reduced Basement Membrane to generate organoids. To perform colonic differentiation, cells were treated with 3 μM CHIR99021 (Cat# 034-23103, FUJIFILM Wako Pure Chemical), 0.5 μM A-83-01 (Cat# 035-24113, FUJIFILM Wako Pure Chemical), 50 ng/mL Noggin (Cat# 120-10C, PeproTech), 30 ng/μL Forskolin (Cat# 063-02193, FUJIFILM Wako Pure Chemical), and 50 ng/mL EGF (Cat# AF-100-15, PeproTech) in advanced DMEM/F12 supplemented with 1× N2 (Cat# 141-08941, FUJIFILM Wako Pure Chemical), 1× B-27 Supplement Minus Vitamin A, 1× GlutaMAX, 0.05% bovine serum albumin, and 1× penicillin−streptomycin for 13 days. To perform the infection experiments, colon organoids were recovered from Matrigel, and the suspension of colon organoids (small free-floating clumps) was seeded onto Matrigel-coated 24-well cell culture plates (1.0 × 10^5^ cells/2 cm^2^) and cultured for 3 days.

#### HEK293 cells

In Figures 2D, 3A–3C, 3G, S3B, S4, and S9, HEK293 cells (Cat# JCRB9068, JCRB Cell Bank) were maintained with Dulbecco’s Modified Eagle Medium (DMEM) with L-Glutamine and Phenol Red (Cat# 044-29765, FUJIFILM Wako Pure Chemical) supplemented with 10% fetal bovine serum (FBS, Cat# 35-010-CV, Corning), 1× GlutaMAX, and 1× penicillin-streptomycin. In Figures 3D–3F, S6, and S7, HEK293 cells (Cat# CRL-1573, ATCC) were maintained with DMEM (high glucose) (Cat# 6429-500ML, Sigma-Aldrich) containing 10% fetal bovine serum (Cat# 172012-500ML, Sigma-Aldrich) and 1% penicillin-streptomycin (Sigma-Aldrich, Cat# P4333-100ML). For cell passaging, HEK293 cells were treated with TrypLE Select Enzyme for 5 min at 37°C.

#### HeLa cells

HeLa cells (Cat# RCB0007, RIKEN BRC) were maintained with DMEM (high glucose) supplemented with 10% FBS, 1× GlutaMAX, and 1× penicillin-streptomycin. For cell passaging, HeLa cells were treated with TrypLE Select Enzyme for 5 min at 37°C.

#### MPXV preparation

Mpox virus (MPXV) infection experiments were performed in a biosafety level 3 (BSL3) facility. Three MPXV strains, clade Ia (Zr-599, Genbank accession no. NC_003310.1), clade IIa (Liberia, Genbank accession no. DQ011156.1), and clade IIb (TKY220091, Genbank accession no. LC722946.1), were propagated using VeroE6 cells (kindly gifted by Tokyo Metropolitan Institute of Public Health, Japan). Zr-599 and Liberia strains were kindly gifted by the National Institute of Infectious Diseases, Japan. TKY220091 was kindly gifted by the Tokyo Metropolitan Institute of Public Health, Japan.

To prepare working MPXV stock, VeroE6 cells (5 ×10^6^ cells) were seeded in a T-75 flask. The next day, the culture medium was changed to DMEM (low glucose) (Cat# D6046-500ML, Sigma-Aldrich) containing 2% FBS and 1× penicillin-streptomycin, and the seed virus was inoculated. At 3 or 4 days post-infection (dpi), the infected flask, including culture supernatant and cells, was frozen at -80°C. Prior to use, the flask was placed at room temperature to facilitate thawing. The thawed medium in the flask was harvested and centrifuged, and the supernatant was collected as a working MPXV stock.

### Method details

#### MPXV titration

In VeroE6 cells, infectious viral titers were determined by plaque assay.^44^ One day before infection, VeroE6 cells (0.2 ×10^6^ cells) were seeded into a 12-well plate and infected with MPXV. Before infection, the culture medium was changed to DMEM (low glucose) containing 2% FBS and 1% penicillin-streptomycin. Cells were incubated at 37°C in a humidified atmosphere of 5% CO_2_. At 3 dpi, the culture medium was removed, and cells were washed with PBS once and fixed with 4% paraformaldehyde phosphate (Cat# 09154-85, Nacalai Tesque). Fixed cells were washed with tap water, dried, and stained with a staining solution (0.1% methylene blue (Cat# 22412-14, Nacalai Tesque) in water) for 30 min. Stained cells were washed with tap water and dried, and the number of plaques was measured to calculate the plaque-forming unit (PFU).

In HEK293 cells, infectious viral titers were determined by focus-forming assay.^45^ One day before infection, HEK293 cells (0.125×10^6^ cells) were seeded into a 24-well plate and infected with MPXV. The virus stock was diluted 10-fold (1:10–1:10^5^) in DMEM containing 10% FBS and 1% penicillin-streptomycin. Cells were incubated at 37°C in a humidified atmosphere of 5% CO_2_. At 3 dpi, the culture medium was removed, and cells were washed with PBS (+) once and fixed with a 10% formaldehyde neutral buffer solution (Cat# 37152-51, Nacalai Tesque) for 20 min. After permeabilization with 1% Triton X-100 (Cat# 35501-15, Nacalai Tesque) in PBS (−) for 5 min, cells were incubated with Mouse anti-Vaccinia virus L1 protein antibody (Cat#65-038, BioAcademia) (×1,000 dilution) at 37°C for 60 min. After washing with PBS (−), cells were incubated with goat anti-mouse IgG (H+L)-HRP (Cat# 5220-0341, KPL) (×3,000 dilution) at 37°C for 60 min. The foci of infected cells were visualized using a Peroxidase Stain 3,3’-diaminobenzidine (DAB) Kit (Cat# 25985-50, Nacalai Tesque) prepared with the Metal Enhancer for DAB Stain (Cat# 07388-24, Nacalai Tesque). Stained cells were washed with tap water and dried, and the number of foci was measured to calculate the focus-forming unit (FFU). The calculated FFU values were used for all infection experiments in HEK293 cells.

#### Quantification of intracellular viral DNA

Total DNA was isolated using DNeasy Blood & Tissue Kit (Cat# 69506, QIAGEN). Viral DNA was quantified using TaqMan Fast Advanced Master Mix for qPCR (Cat# 4444557, Thermo Fisher Scientific) with 5’-GGA AAA TGT AAA GAC AAC GAA TAC AG-3’ as the forward primer, 5’-GCT ATC ACA TAA TCT GGA AGC GTA-3’ as the reverse primer, and 5’-FAM-AAG CCG TAA TCT ATG TTG TCT ATC GTG TCC-BHQ1-3’ as the probe.^34^ To quantify viral DNA copy numbers, a plasmid encoding the *MPXV G2R* gene was used as the standard.

#### Quantitative PCR

Total RNA was isolated using ISOGEN (Cat# 319-90211, NIPPON GENE). cDNA was synthesized using 500 ng of total RNA with the Superscript VILO cDNA Synthesis Kit (Cat# 11754250, Thermo Fisher Scientific). Real-time RT-qPCR was performed with the SYBR Green PCR Master Mix (Cat# 4385614, Thermo Fisher Scientific) using a StepOnePlus real-time PCR system (Thermo Fisher Scientific) or QuantStudio 3 real-time PCR system (Thermo Fisher Scientific). The relative quantification of target mRNA levels was performed using the 2^-ΔΔCT^ method. Values were normalized to the housekeeping gene *glyceraldehyde 3-phosphate dehydrogenase* (*GAPDH*). Primer sequences are summarized in Table S1.

#### SOD activity assay

HEK293 cells (2×10^6^ cells) were seeded in 100 mm dishes and cultured for 2 days. For overexpressing OPG175 or human superoxide dismutase (hSOD)1, pRP[Exp]-CBh>[MPXV I gp152], pRP[Exp]-CBh>[MPXV IIb BDQ10531.1], and pRP[Exp]-CBh>hSOD1 were constructed by VectorBuilder Inc. Vector IDs are VB230516-1089snp, VB230516-1094nak, and VB230516-1107bkv, respectively. As a control vector, we used pRP[Exp]-CBh>ORF_Stuffer (vector ID: VB230622-1540udv). These plasmid vectors were transfected using Lipofectamine 2000 (Cat# 11668019, Thermo Fischer Scientific) and cultured for 2 days. To evaluate transfection efficiency in HEK293 cells, pCMV-EGFP plasmids (NEPA GENE) were transfected using Lipofectamine 2000 and cultured for 2 days. GFP fluorescence was observed using IX83 (Evident Corporation). Transfected cells were collected by TrypLE Select and suspended in 1 mL of PBS. Cells were homogenized by freeze-thawing, centrifuged at 10,000 × *g* for 15 min, and the supernatants were used as assay samples. The SOD activity was measured using the SOD Assay Kit - WST (Cat# S311, DOJINDO LABORATORIES) according to the manufacturer’s instructions. The units of SOD were calculated using the following equation:

Units (U/10^7^ cells) = 1/IC_50_ ÷ Sample volume ÷ Number of cells (×10^7^)

#### Establishment of shRNA-expressing HEK293 cells using *piggyBac* system

The sequence of shRNA against OPG175 was designed using siDirect.^35^^,^^36^ For expressing shRNA, pPB[shRNA]-Bsd-U6>[clade_IIb_OPG175_105-127], pPB[shRNA]-Hygro-U6>[clade_IIb_OPG175_215-237], and pPB[shRNA]-Puro-U6>[clade_IIb_OPG175_239-261] were constructed by VectorBuilder Inc (Table S2). The vector IDs are VB231109-1138pwv, VB231109-1137pdb, and VB231109-1136hjg, respectively. The plasmid vector expressing PBase, pHL-EF1a-hcPBase, was kindly gifted by Dr. Akitsu Hotta (Kyoto University). These plasmids were electroporated into HEK293 cells using the 4D-Nucleofector system (Lonza). Three days after electroporation, HEK293 cells in which the three shRNA constructs were inserted were selected using 10 μg/mL blasticidin S (Cat# 03759-71, Thermo Fisher Scientific), 50 μg/mL hygromycin B (Cat# 09287-84, Nacalai Tesque), and 10 μg/mL puromycin (Cat# 14861-71, Thermo Fisher Scientific).

#### MPXV infection experiments using shRNA-expressing HEK293 cells

Control HEK293 cells or shRNA-expressing HEK293 cells were seeded on collagen I-coated 96-well plates (Cat# 4860-010, AGC TECHNO GLASS) at 2×10^4^ cells/well and cultured for 1 day. Cells were infected with MPXV clade Ia at 0.05 multiplicity of infection (MOI) for 2 hours, then the medium was changed, and cells were cultured for 4 days.

#### MPXV infection experiments using OPG175-overexpressed HEK293 cells

HEK293 cells were seeded on Poly-D-Lysine-coated 96-well plates (Cat# 356461, Corning) at 4×10^4^ cells/well and cultured for 1 day. The cells were transfected with the plasmid vector expressing OPG175 (pRP-CBh-OPG175; Vector ID: VB230516-1089snp) or control plasmid (pRP-CBh-control; Vector ID: VB230622-1540udv) using Lipofectamine 2000 and cultured for 1 day. The plasmid vectors were constructed by VectorBuilder Inc. Cells were infected with MPXV clade Ia at 0.05 MOI for 2 hours, then the medium was changed, and cells were cultured for 2 days.

#### MPXV infection experiments using siOPG175-transfected HeLa cells

HeLa cells were seeded on 96-well plates at 2×10^4^ cells/well and cultured for 1 day. The cells were transfected with 50 nM siRNA targeting OPG175 (siOPG175; DsiRNA, Integrated DNA Technologies) or control siRNA (siControl; Negative Control DsiRNA, Cat# 51-01-14-04, Integrated DNA Technologies) using Lipofectamine RNAiMAX (Cat# 13778150, Thermo Fisher Scientific) and cultured for 4 hours. siOPG175 was prepared by pooling three siRNAs (Table S3) targeting different sequences of the *OPG175* gene. Cells were infected with MPXV clade IIb at 0.05 MOI for 2 hours, then the medium was changed, and cells were cultured for 2 days.

#### OPG175 and hSOD1 overexpression

The plasmid vector expressing OPG175 or hSOD1 was transfected into HEK293 cells using PEI Max (Cat# 24765-1, Polysciences). Two days after transfection, total RNA was isolated from cells and performed RNA-seq analysis.

#### BIO treatment

HEK293 cells were seeded on collagen I-coated 96-well plates at 2×10^4^ cells/well and cultured for 1 day. The culture medium was changed to DMEM containing 6-bromoindirubin-3'-oxime (BIO, Cat# 361550, Sigma-Aldrich) or 0.5% DMSO (Nacalai Tesque, Cat# 13408-64), then cells were infected with MPXV clade Ia, IIa, or IIb at 0.05 MOI. Two hours after infection, the culture medium was changed with DMEM containing BIO or 0.5% DMSO.

For the cytotoxicity assay of BIO, HEK293 cells were seeded on collagen I-coated 96-well plates at 2×10^4^ cells/well and cultured for 1 day. The culture medium was changed with DMEM containing BIO, and cells were cultured for 4 days. The cytotoxicity of BIO was evaluated using the Cell Counting Kit-8 (Cat# 347-07621, FUJIFILM Wako Pure Chemical) according to the manufacturer’s instructions.

#### Wnt3A-conditioned medium

Wnt3A-conditioned medium (Wnt-CM) was collected using L-Wnt-3A cells (Cat# CRL-2647, ATCC). L-Wnt-3A cells were cultured in DMEM supplemented with 10% FBS, 1× GlutaMAX, and 1× penicillin-streptomycin, filtered through a 0.22 μm filter, and stored at -80°C. Wnt3A-conditioned medium was mixed 1:1 with DMEM supplemented with 10% FBS, 1× GlutaMAX, and 1× penicillin-streptomycin, and then HEK293 cells were treated with Wnt3A-conditioned medium.

For MPXV infection experiments, HEK293 cells were seeded on collagen I-coated 96-well plates at 2×10^4^ cells/well and cultured for 1 day. The culture medium was changed to Wnt-CM or DMEM, then cells were infected with MPXV clade Ia at 0.05 MOI. Two hours after infection, the culture medium was changed with Wnt-CM or DMEM.

#### RNA sequencing

Total RNA was isolated from cells using ISOGEN. RNA integrity was assessed using a 2100 Bioanalyzer (Agilent Technologies). Library preparation was performed using an Illumina Stranded mRNA Prep Kit (Cat# 20040532, Illumina) according to the manufacturer’s instructions. Sequencing was performed on an Illumina NextSeq2000. The fastq files were generated using bcl2fastq-2.20. Adapter sequences and low-quality bases were trimmed from the raw reads using Cutadapt ver v4.6.^37^ For the transcriptome analysis of human, the trimmed reads were mapped to human reference genome sequences (hg38) using STAR ver 2.7.11a^38^ with the GENCODE (release 32, GRCh38.p13) gtf file.^33^ For the transcriptome analysis of MPXV, the trimmed reads were mapped to MPXV reference genome sequences of each clade using STAR ver 2.7.11a with these gtf file. The raw counts were calculated using htseq-count ver. 2.0.5^39^ with the GENCODE gtf file. Differential gene expression analysis was performed using DESeq2 v1.42.0^40^ with the raw count data. For visualization and general comparison of gene expression levels, Transcripts Per Kilobase Million (TPM) values were calculated. The differentially expressed genes were visualized as volcano plots using the “EnhancedVolcano” function of the “EnhancedVolcano” package. Gene ontology enrichment analysis was performed using “enrichGO” function of the “clusterProfiler” package.^41^^,^^42^ The dot plot showing the result of GO enrichment analysis was generated by “dotplot” function of the “enrichplot” package. Raw data generated from OPG175 and hSOD1 overexpression experiments were submitted under Gene Expression Omnibus (GEO) accession number GSE278866. RNA-seq data of MPXV-infected keratinocytes were obtained from our previous reports (GSE219036).

The comparison of viral gene expression was performed by the following procedure. First, a list of amino acid sequences of proteins contained in the MPXV of each clade was downloaded in FASTA format from NCBI genes. Next, an all-to-all pairwise alignment was performed between clades Ia and IIa, or IIa and IIb, for the amino acid sequences in the list using the “pairwiseAlignment” function of the “Biostrings” package. The alignment “Score” values were visualized as heatmaps using the “Heatmap” function of the “ComplexHeatmap” package.^43^ The number of genes conserved among clades was counted based on the heatmaps and visualized as a Venn diagram using the “venn.diagram” function of the “VennDiagram” package. For genes conserved in all three clades, the average TPM in MPXV clade IIb was calculated from the average TPM in MPXV clades Ia and IIa for genes conserved in the three MPXV clades.

### Quantification and statistical analysis

Statistical significance was evaluated using Student’s *t*-test, or one-way analysis of variance (ANOVA) followed by Tukey’s or Dunnett’s post hoc tests using GraphPad Prism 9. Data are representative of three independent experiments unless otherwise described. Details are described in the figure legends.
