# Supplementary material for: Mpox virus OPG175 negatively regulates viral replication by controlling Wnt signaling
Source: iScience. 2025 Nov 19;28(12):114105. doi: 10.1016/j.isci.2025.114105 (PMC12756559; doi:10.1016/j.isci.2025.114105)
Supplement: Document S1. Figures S1–S9 and Tables S1–S3 [file mmc1.pdf]

iScience, Volume *28*

Supplemental information

Mpox virus OPG175 negatively regulates viral replication by controlling Wnt signaling

Yoshitaka Nakata, Masako Yamasaki, Yukio Watanabe, Keiya Uriu, Rina Hashimoto, Takuya Yamamoto, The Genotype to Phenotype Japan (G2P-Japan) Consortium, Kei Sato, Akatsuki Saito, and Kazuo Takayama

Figure S1

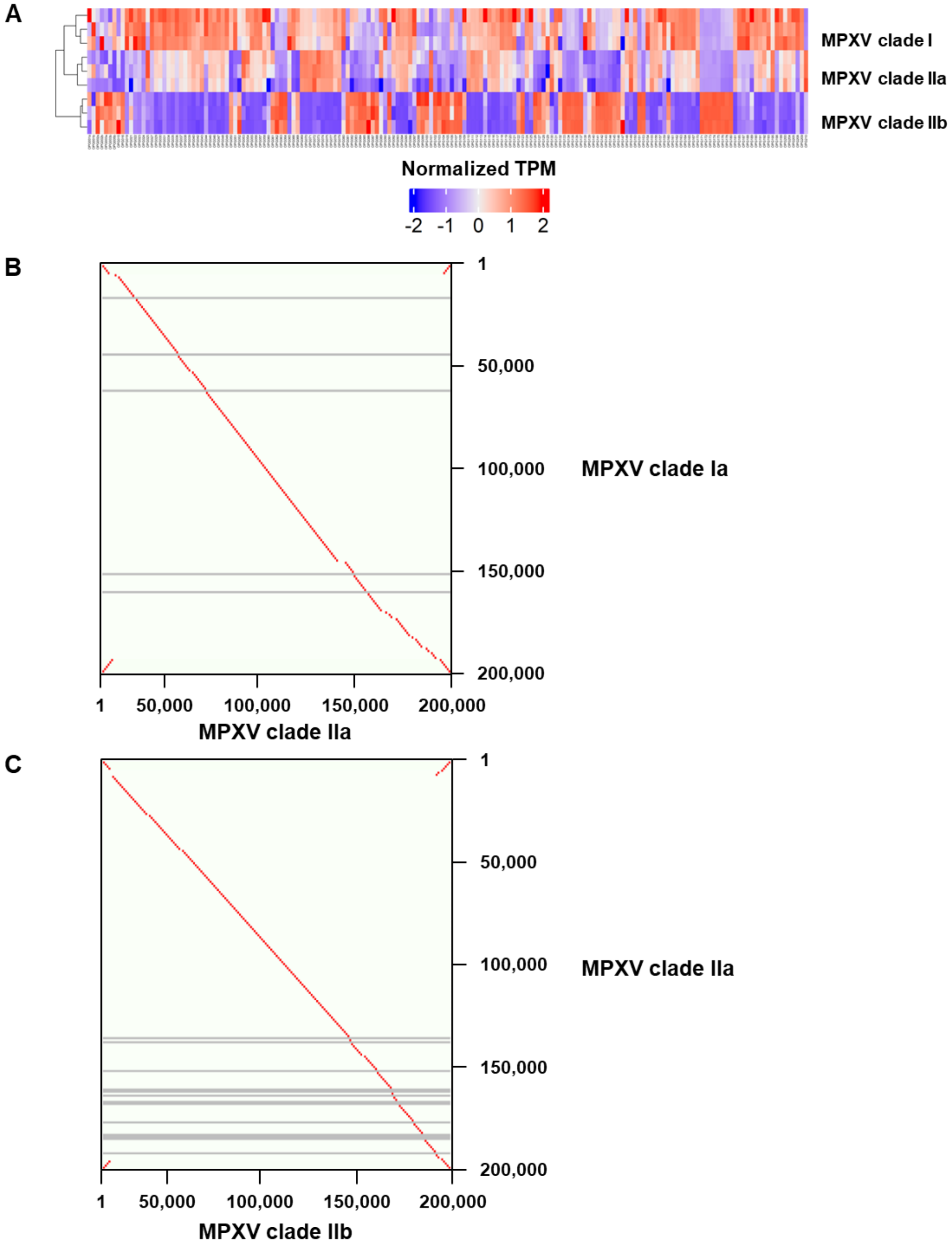

Figure S1. Expression profile of MPXV genes, Related to Figure 1

(**A**) Heatmap showing the expression profile of MPXV genes. TPM values of each MPXV gene were visualized and clustered. (**B, C**) Dot plots representing the conservation of amino acid sequences between MPXV clades Ia and IIa (**B**) or MPXV clades IIa and IIb

(**C**). Pairwise alignment was performed, with alignment scores visualized as a heat map. The vertical and horizontal axes represent the approximate position of each protein in the MPXV genome.

Figure S2

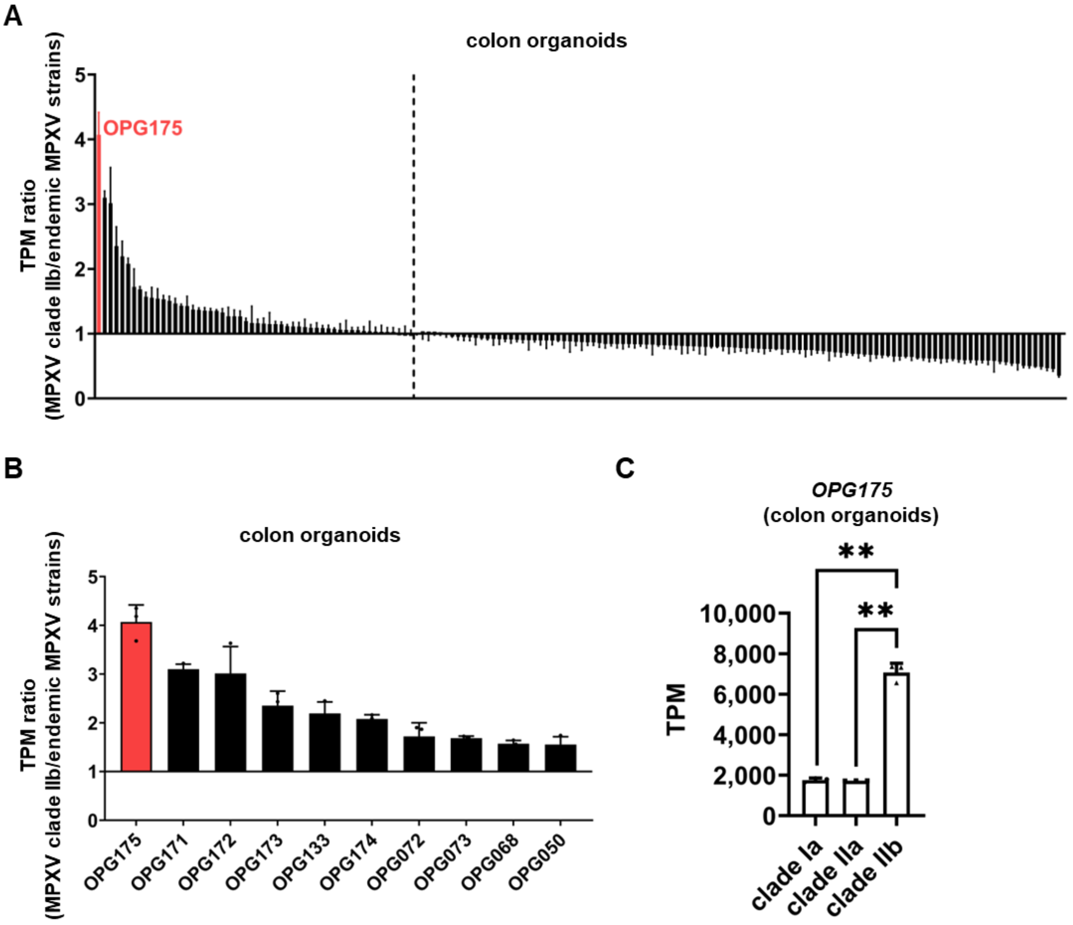

Figure S2. OPG175 is highly expressed in MPXV clade IIb-infected colon organoids, Related to Figure 1

(**A, B**) Ratios of all (**A**) or the top 10 (**B**) MPXV genes in MPXV-infected colon organoids. The ratio of TPM values of viral genes in MPXV clade IIb- to endemic strain-infected cells is shown. Data are shown as mean+SD (*n*=3). (**C**) TPM values of *MPXV OPG175* in MPXV clade Ia-, MPXV clade IIa-, or MPXV clade IIb-infected colon organoids. One-way ANOVA followed by Tukey post hoc test (**$p < 0.01$). Data are shown as mean+SD (*n*=3).

Figure S3

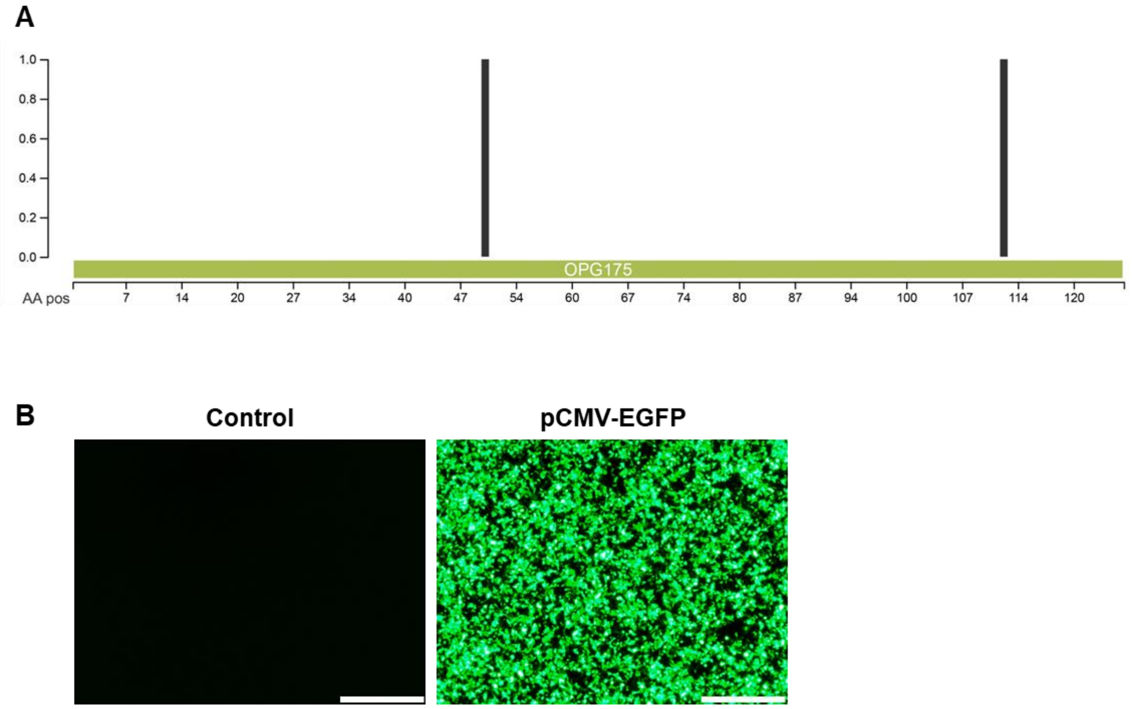

Figure S3. The amino acid sequence of OPG175 is conserved among clade IIb MPXV and transfection efficiency of HEK293 cells, Related to Figure 2

(**A**) Bar plot representing amino acid diversity of MPXV OPG175 from the Nextstrain mpox website (https://nextstrain.org/mpox/all-clades?c=gt-nuc_148344). The vertical axis shows the frequency of amino acid substitution. (**B**) Fluorescence images of control and GFP-transfected HEK293 cells. HEK293 cells were transfected with GFP-expressing plasmid vectors (pCMV-EGFP). The cells were analyzed 48 hours after transfection using IX83. Scale bars = 50 µm.

Figure S4

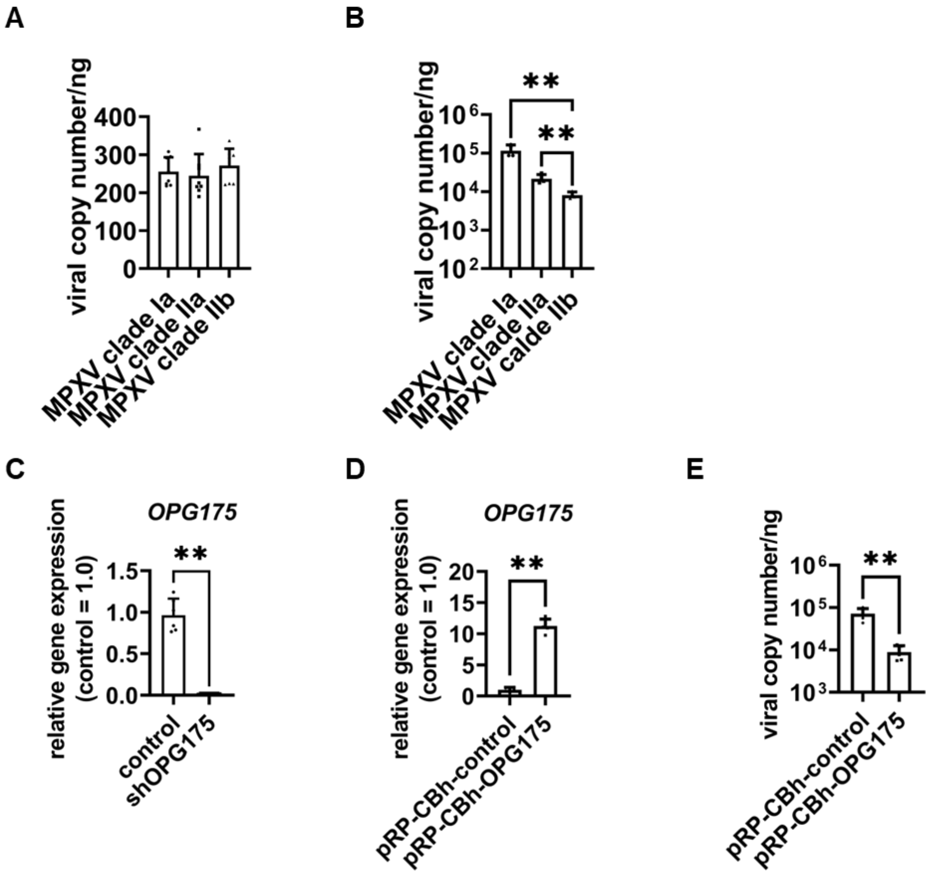

Figure S4. Intracellular viral DNA in the cells infected with each MPXV clades, and the impact of OPG175 expression on MPXV replication, Related to Figure 3

(**A**) HEK293 cells were infected with MPXV clade Ia, IIa, or IIb at 0.05 multiplicity of infection (MOI). The intracellular viral DNA was measured at 2 hpi. One-way ANOVA followed by Tukey's post hoc test. Data are shown as mean+SD (*n*=8). (**B**) HEK293 cells were infected with MPXV clade Ia, IIa, or IIb at 0.05 MOI, and then cultured for 96 hours. The intracellular viral DNA in the HEK293 cells were measured by qPCR at 96 hpi. One-way ANOVA followed by Dunnett post hoc test (***p* < 0.01, MPXV clade IIb versus other groups). Data are shown as mean+SD (*n*=4). **(C)** Wild type (control) or shOPG175-expressing HEK293 cells (shOPG175) were infected with MPXV clade Ia at 0.05 MOI and cultured for 48 hours. The expression of *MPXV OPG175* was measured by RT-qPCR. Unpaired two-tailed Student's *t*-tests (***p* < 0.01). Data are shown as mean+SD (*n*=6). (**D,E**) HEK293 cells were transfected with control plasmids (pRP-CBh-control) or OPG175-expressing plasmids (pRP-CBh-OPG175). One day after transfection, the cells were infected with MPXV clade Ia at 0.05 MOI, and then cultured for 48 hours. The expression of *MPXV OPG175* was measured by RT-qPCR (**D**). The intracellular viral

DNA was measured by qPCR (**E**). Unpaired two-tailed Student's *t*-tests (**$p < 0.01$). Data are shown as mean+SD (n=5).

Figure S5

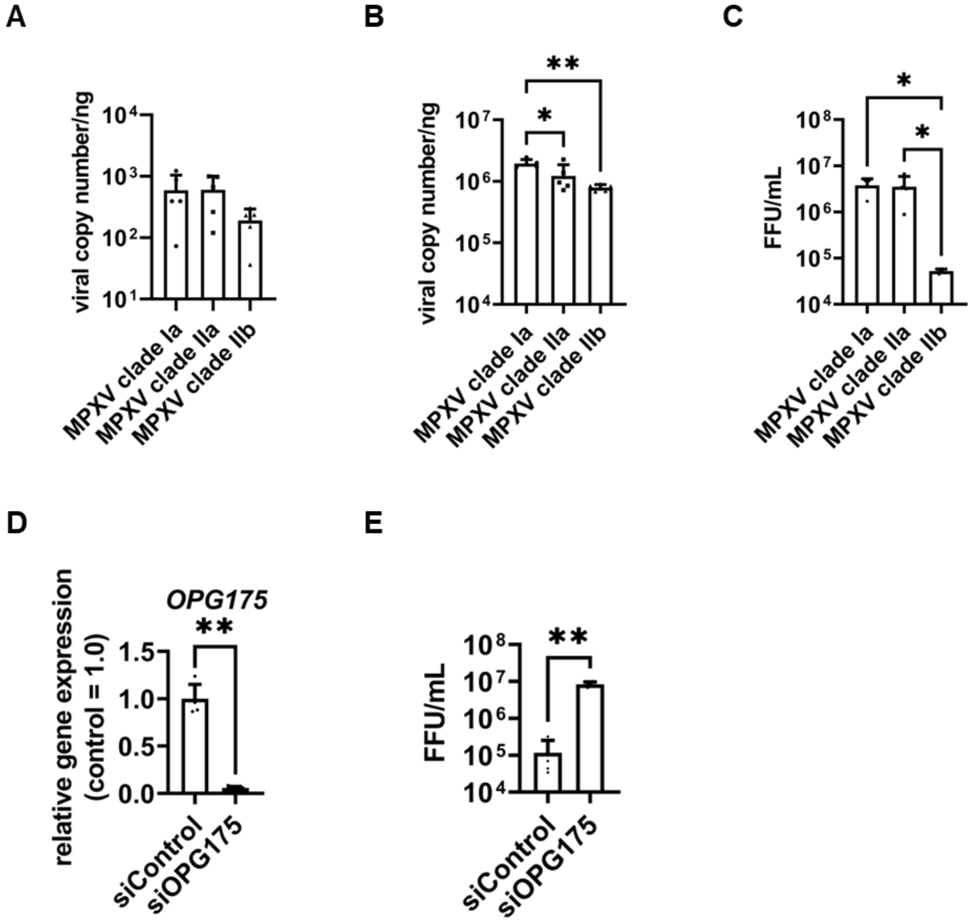

Figure S5. MPXV infection experiments using HeLa cells, Related to Figure 3

(**A**) HeLa cells were infected with MPXV clade Ia, IIa, or IIb at 0.05 MOI. The intracellular viral DNA was measured at 2 hpi. One-way ANOVA followed by Tukey's post hoc test. Data are shown as mean+SD (n=5). (**B**) HeLa cells were infected with MPXV clade Ia, IIa, or IIb at 0.05 MOI, and then cultured for 96 hours. The intracellular viral DNA in the HeLa cells was measured by qPCR at 96 hpi. One-way ANOVA followed by Tukey's post hoc test (*p<0.05, **$p < 0.01$). Data are shown as mean+SD (n=5). (**C**) HeLa cells were infected with MPXV clade Ia, IIa, or IIb at 0.05 MOI, and then cultured for 96 hours. The FFU values were measured at 96 hpi. One-way ANOVA followed by Dunnett post hoc test (*$p < 0.05$, MPXV clade IIb versus other groups). Data are shown as mean+SD (n=4). (**D**) HeLa cells were transfected with control siRNA (siControl) or OPG175-targeting siRNA (siOPG175). Four hours after transfection, the cells were infected with MPXV clade IIb at 0.05 MOI, and then cultured for 48 hours. The expression of *MPXV OPG175* was measured by RT-qPCR. Unpaired two-tailed Student's t-tests (**$p < 0.01$). Data are shown as mean+SD (n=5). (**E**) HeLa cells were

transfected with siControl or siOPG175. Four hours after transfection, the cells were infected with MPXV clade IIb at 0.05 MOI and cultured for 48 hours. The FFU values were measured at 48 hpi. Unpaired two-tailed Student's *t*-tests (**$p < 0.01$). Data are shown as mean+SD (n=4).

Figure S6

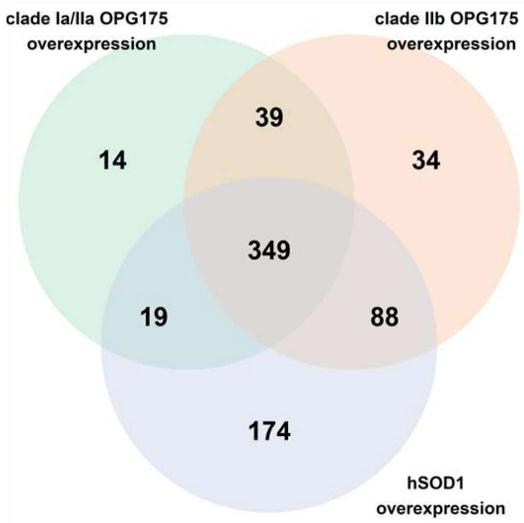

Figure S6. Genes upregulated by OPG175 or hSOD1 overexpression, Related to Figure 3

HEK293 cells were transfected with control, OPG175-, or hSOD1-expressing plasmids and cultured for 48 hours. RNA-seq analysis was performed. Venn diagram representing the number of genes upregulated in each condition.

Figure S7

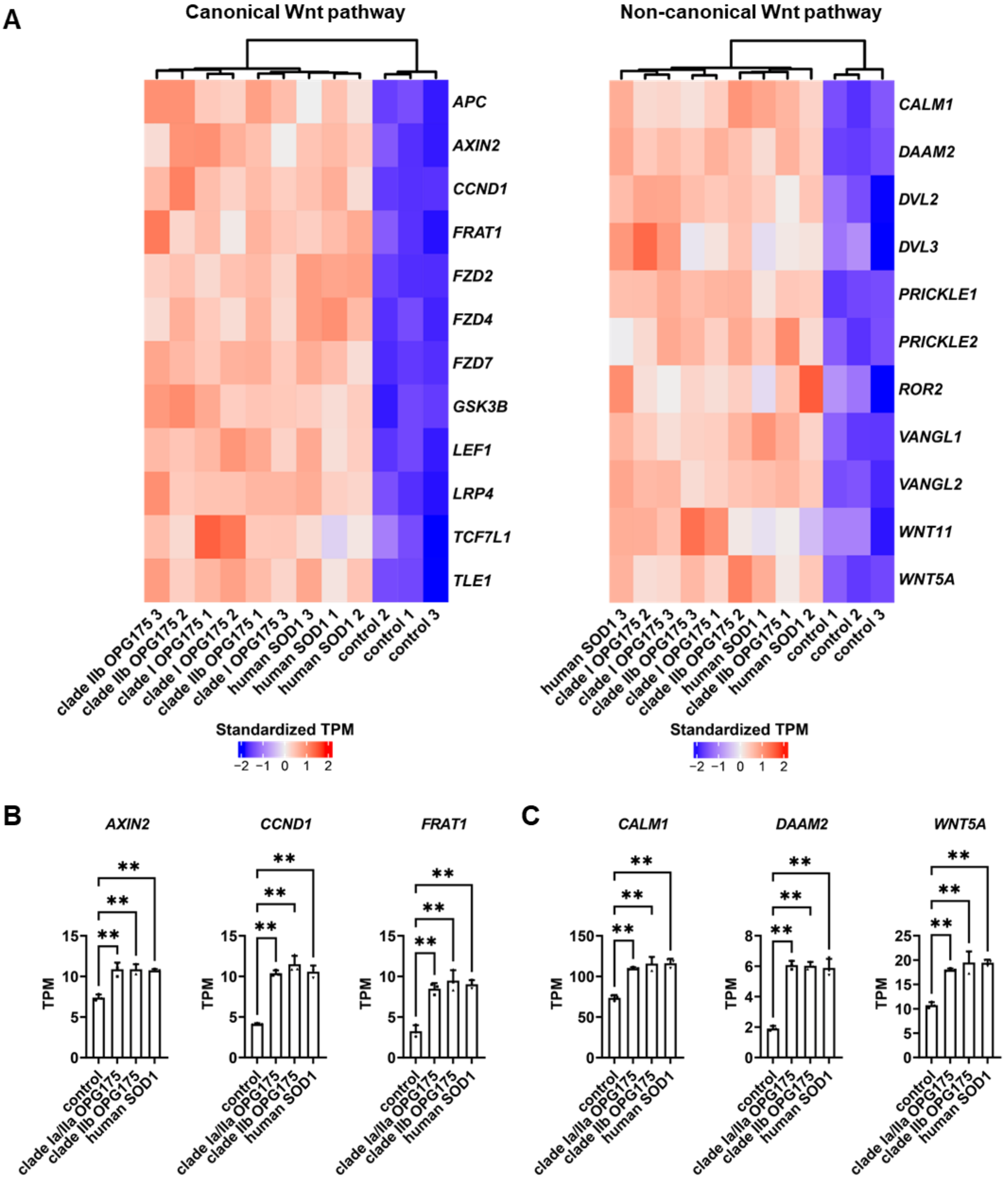

Figure S7. Expression levels of Wnt signaling-related genes were upregulated in MPXV-infected keratinocytes, Related to Figure 3

(**A**) Heatmap showing the expression levels of canonical or non-canonical Wnt pathway-related genes. TPM values of each gene were visualized and clustered. (**B**) TPM values of *axin 2* (*AXIN2*), *cyclin D1* (*CCND1*), and *FRAT regulator of WNT signaling pathway 1* (*FRAT1*) in OPG175- or hSOD1-overexpressed HEK293 cells. One-way ANOVA followed by Dunnett post hoc test (**$p < 0.01$, control versus other groups). Data are shown as mean+SD (n=3). (**C**) TPM values of *calmodulin 1* (*CALM1*), *dishevelled*

associated activator of morphogenesis 2 (*DAAM2*), and *Wnt family member 5A* (*WNT5A*) in OPG175- or hSOD1-oveexpressed HEK293 cells. One-way ANOVA followed by Dunnett post hoc test (**$p < 0.01$, control versus other groups). Data are shown as mean+SD (n=3).

Figure S8

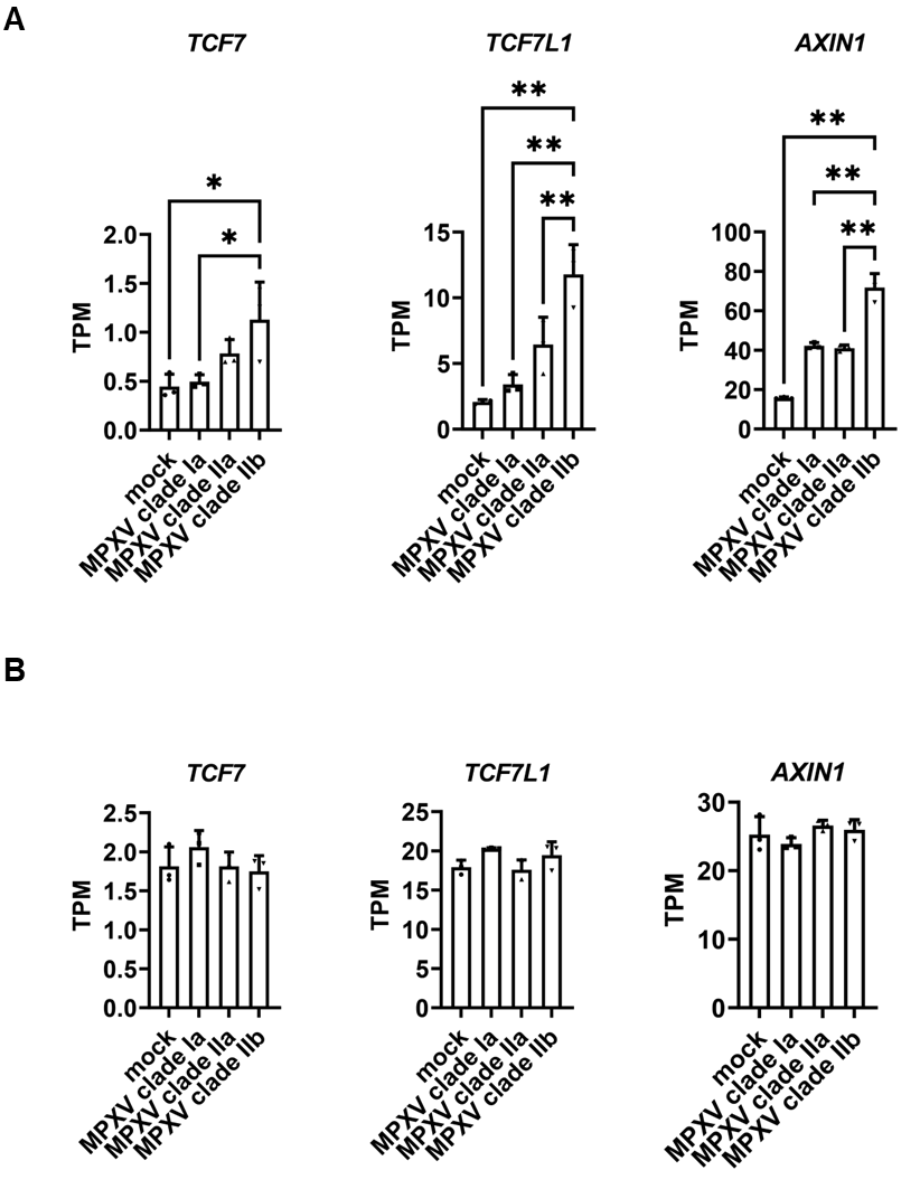

Figure S8. Expression levels of Wnt signaling-related genes were upregulated in MPXV-infected keratinocytes, Related to Figure 3

(**A, B**) Keratinocytes or colon organoids were infected with MPXV clade Ia, IIa, or IIb at 0.05 MOI and cultured for 72 hours. RNA-seq analysis was performed. TPM values of canonical Wnt pathway-related genes (*Transcription factor 7 (TCF7), Transcription factor 7-like 1 (TCF7L1), and Axin-1 (AXIN1)*) in keratinocytes (**A**) and colon organoids (**B**). One-way ANOVA followed by Dunnett post hoc test (*$p<0.05$, **$p < 0.01$, MPXV clade IIb versus other groups). Data are shown as mean+SD (n=3).

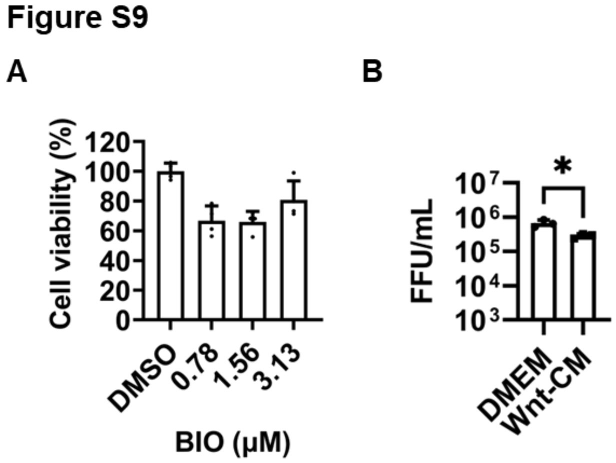

Figure S9. Cell viability measurement in BIO treatment experiment and MPXV infection experiment with Wnt3A-conditioned medium treatment, Related to Figure 3

(**A**) HEK293 cells were treated with BIO-containing medium for 96 hours, and cell viability was measured using the Cell Counting Kit-8. Data are shown as mean+SD (n=4). (**B**) HEK293 cells were infected with MPXV clade Ia at 0.05 MOI and cultured with DMEM or Wnt3A-conditioned medium (Wnt-CM) for 96 hours. The FFU values were measured at 96 hpi. Unpaired two-tailed Student's t-tests ($*p < 0.05$). Data are shown as mean+SD (n=3).

Table S1. Primers used for RT-qPCR, Related to STAR Methods

Gene name	Forward primer	Reverse primer
GAPDH	GGAGCGAGATCCCTCCAAAAT	GGCTGTTGTCATACTTCTCATGG
OPG175	TCGAGGATGTGATTCCATAGG	AAACGCCATTCTCGTTAATTGT

Table S2. shRNA-OPG175 used for knockdown experiments, Related to STAR Methods

shRNA	Vector ID	Target sequences (5'-3')
1	VB231109-1138pwv	CGGAACGTATAGTTTGATAATTC
2	VB231109-1137pdb	TAGCATATGTTTATTTAGATACA
3	VB231109-1136hjg	ATGTAAATATATCTACAATTATT

Table S3. siRNA-OPG175 used for knockdown experiments, Related to STAR Methods

siRNA	Strand	Sequences (5’-3’)
1	Sense	rGrGrArArArGrGrCrGrUrUrArUrCrUrArUrUrUrCrArArAAA
	Antisense	rUrUrUrUrUrGrArArArUrArGrArUrArArCrGrCrCrUrUrUrCrCrArA
2	Sense	rCrCrArGrArArArUrArUrUrUrArUrCrGrGrUrArArCrArUCT
	Antisense	rArGrArUrGrUrUrArCrCrGrArUrArArArUrArUrUrUrCrUrGrGrArC
3	Sense	rArCrGrGrArGrArUrArUrUrArGrUrCrGrArGrGrArUrGrUGA
	Antisense	rUrCrArCrArUrCrCrUrCrGrArCrUrArArUrArUrCrUrCrCrGrUrArA
